# Albuminuria as the foundation for modern nephroprotective therapy

**DOI:** 10.1590/2175-8239-JBN-2026-0250en

**Published:** 2026-09-07

**Authors:** Guilherme Travaini Chinalia, Bruno dos Santos Scarabello

**Affiliations:** 1 Universidade de Ribeirão Preto, Faculdade de Medicina, Ribeirão Preto, SP, Brazil.

Dear Editor,

We read with great interest the article by Moraes et al.^1^, which evaluated strategies for screening chronic kidney disease (CKD) in individuals with diabetes mellitus (DM) and hypertension without a previous diagnosis of kidney disease, highlighting undiagnosed cases encountered in routine clinical practice. The authors also discussed the underutilization of albuminuria as a risk stratification tool. However, we would like to complement this discussion from an additional perspective: beyond its relevance for early diagnosis, the appropriate assessment of albuminuria has become central to the implementation of therapeutic strategies capable of modifying the natural history of CKD. In this context, early detection extends beyond disease identification and enables the timely initiation of modern nephroprotective therapies, such as sodium-glucose cotransporter 2 inhibitors (SGLT2i) and glucagon-like peptide-1 receptor agonists (GLP-1RAs), whose use should be guided by the appropriate assessment of renal and cardiovascular risk^2^.

Early recognition of CKD has become even more relevant in light of recent advances in available therapeutic strategies. Among these, SGLT2 inhibitors stand out for restoring tubuloglomerular feedback and reducing glomerular hyperfiltration—one of the main mechanisms involved in the progression of kidney injury in individuals with DM. This hemodynamic effect, together with reductions in albuminuria and the preservation of the glomerular filtration rate over time, contributes to slowing CKD progression and reducing cardiovascular events^2,3^. In addition to reflecting the risk of CKD progression, albuminuria has become a key marker for identifying patients most likely to benefit from nephroprotective therapies. Consequently, its systematic assessment has become essential for individualized treatment and the timely implementation of these interventions^2^. Thus, the early identification of eligible patients is no longer solely a diagnostic objective but also a crucial step toward the timely implementation of interventions capable of modifying disease progression.

In this regard, the importance of early CKD detection extends beyond diagnosis and has direct implications for therapeutic decision-making. This paradigm shift is reflected in contemporary clinical guidelines, which recommend combined stratification using estimated glomerular filtration rate (eGFR) and albuminuria to identify patients at higher risk of CKD progression and to guide the initiation of nephroprotective therapies, particularly SGLT2 inhibitors^2^. More recently, accumulating evidence has also demonstrated renal and cardiovascular benefits associated with GLP-1RAs, including reductions in albuminuria progression and the slowing of kidney function decline among high-risk populations^4^. Therefore, the underutilization of albuminuria should not be viewed merely as a diagnostic limitation but also as a potential barrier to timely access to therapies capable of improving clinically relevant outcomes^3,4^.

Accordingly, expanding the systematic use of albuminuria not only enhances CKD screening but also facilitates the timely implementation of therapies capable of modifying its natural course. In this context, the routine incorporation of albuminuria into the assessment of patients with DM and hypertension represents both a diagnostic strategy and an essential component of contemporary nephroprotective care.
