# Supplementary material for: Albuminuria as the foundation for modern nephroprotective therapy
Source: J Bras Nefrol. 2026 Sep 7;48(4):e20260250. doi: 10.1590/2175-8239-JBN-2026-0250en (PMC13551828; doi:10.1590/2175-8239-JBN-2026-0250en)
Supplement: Translation

# Translation: Albuminuria como base da terapia nefroprotetora moderna

**DOI:** 10.1590/2175-8239-JBN-2026-0250pt

**Authors:** Guilherme Travaini Chinalia, Bruno dos Santos Scarabello

**Affiliations:** 1 Universidade de Ribeirão Preto, Faculdade de Medicina, Ribeirão Preto, SP, Brasil.

Prezado editor,

Lemos com grande interesse o artigo de Moraes et al.^1^, que avaliou estratégias de rastreamento de doença renal crônica (DRC) em indivíduos com diabetes mellitus (DM) e hipertensão arterial sem diagnóstico prévio de comprometimento renal, destacando casos não identificados na prática clínica. Os autores também discutem a subutilização da albuminúria como ferramenta de estratificação de risco. No entanto, gostaríamos de complementar essa discussão a partir de uma perspectiva adicional: além de sua relevância para o diagnóstico precoce, a identificação adequada da albuminúria assume um papel central na definição de estratégias terapêuticas capazes de modificar a história natural da DRC. Nesse cenário, a detecção precoce não se limita à identificação da doença, mas se estende à possibilidade de implementação oportuna de terapias nefroprotetoras modernas, como inibidores do cotransportador sódio-glicose tipo 2 (SGLT2i) e agonistas do receptor do peptídeo semelhante ao glucagon-1 (GLP-1RAs), cuja utilização depende da adequada estratificação do risco renal e cardiovascular^2^.

O reconhecimento precoce da DRC adquire ainda maior relevância diante da evolução recente das estratégias terapêuticas disponíveis. Entre elas, os SGLT2i destacam-se por restaurarem o feedback túbulo-glomerular, reduzindo a hiperfiltração glomerular — um dos principais mecanismos envolvidos na progressão da lesão renal em indivíduos com DM. Esse efeito hemodinâmico, aliado à redução da albuminúria e à preservação da taxa de filtração glomerular ao longo do tempo, contribui para retardar a progressão da DRC e reduzir eventos cardiovasculares^2,3^. Além de refletir o risco de progressão da DRC, a albuminúria passou a desempenhar papel determinante na identificação de pacientes que mais se beneficiam das terapias nefroprotetoras. Assim, sua avaliação sistemática torna-se um componente essencial para a individualização do tratamento e para a implementação dessas intervenções^2^. Dessa forma, a identificação precoce de pacientes elegíveis deixa de representar apenas um objetivo diagnóstico e passa a constituir um passo essencial para a adoção oportuna de intervenções capazes de modificar a evolução da doença.

Nesse contexto, a relevância da detecção precoce da DRC ultrapassa a dimensão diagnóstica e passa a ter implicações diretas na tomada de decisão terapêutica. Essa mudança de paradigma terapêutico também se reflete nas diretrizes contemporâneas, que recomendam a estratificação conjunta pela taxa de filtração glomerular (TFG) e pela albuminúria para identificar pacientes com maior risco de progressão da DRC e orientar a indicação de terapias nefroprotetoras, especialmente os SGLT2i^2^. Mais recentemente, evidências também têm demonstrado benefícios renais e cardiovasculares associados aos GLP-1RAs, incluindo a redução da progressão da albuminúria e a desaceleração da queda da função renal em populações de alto risco.^4^ Assim, a subutilização da albuminúria não deve ser interpretada apenas como uma limitação diagnóstica, mas como um fator potencialmente limitante ao acesso oportuno a terapias capazes de modificar desfechos clínicos relevantes^3,4^.

Dessa forma, ampliar o uso sistemático da albuminúria significa não apenas aprimorar o rastreamento da DRC, mas também viabilizar a implementação de terapias capazes de modificar sua história natural. Nesse contexto, a incorporação sistemática da albuminúria à avaliação de pacientes com DM e hipertensão representa não só uma estratégia diagnóstica, como também um componente essencial da medicina nefroprotetora contemporânea.
